# How Small-Scale Farmers Understand Rain Water Harvesting Technology? Evidence from Northern Ethiopia

**DOI:** 10.1155/2021/8617098

**Published:** 2021-01-28

**Authors:** Abay Tafere Mengistu

**Affiliations:** College of Dry Land Agriculture and Natural Resources, Mekelle University, P.O. Box 231, Mekelle, Ethiopia

## Abstract

The study examined farmers' perceptions and adoption of rain water harvesting technology in Raya-Alamata district of Ethiopia. Four kebeles were purposively selected from the 13 homogeneous kebeles (small administrative unit). During the survey, primary data were collected through a semistructured questionnaire distributed to 270 systematically selected sample respondents and through interview with key informants, development agents, and local administrators. Secondary data were retrieved from district agriculture office, books, and published scientific materials. Descriptive statistics, simple narration, and a probit regression model were used to analyze the data. The results showed that the farmers perceived rain water harvesting as a motivational way of creating sense of belongingness. They think that it increases crop production, increases forest regeneration, and encourages forage production. The probit regression models revealed that determinants of farmers' adoption of rainwater harvesting technology were significantly and positively affected by education, farm size, and off-farm income. The effect of distance to the farmland and farmers training center (FTC) was also significant but negative. Men farmers had higher level of adoption compared to their counterparts. Productive and reproductive roles constrain women household farmers from using the technologies. Based on the finding, the policy implications were as follows: dissemination of information related to rain water harvesting technology should be imperative through formal and informal education. Alternative sources of water need also be promoted. More importantly, female-headed households need to be encouraged to be community leaders to foster the adoption of rain water harvesting technology.

## 1. Introduction

Subsistence rain-fed agriculture is the mainstay of most sub-Saharan African economies and contributes 10–70% to their GDP [1]. African agriculture has the lowest rate of productivity increase in the world. Africa was the only major region with a decline in per capita food production in the years 1980–2000 [2, 3].

Globally, Ethiopia is known as the center of drought and famine. Over 90% of the food supply comes from rain-fed small-holder agriculture, and rainfall failure means loss of major food supply which always results in massive food deficit [4]. The rainfall pattern of the country also shows high level of variations [5]. In the semiarid areas, rainwater is available in abundance during the rainy season and surpasses the evapotranspiration during the three months between July and September [6]. The rain is very poorly distributed in both spatial and temporal terms. Often, there is too much water during a few days of the year, while water supply is insufficient during most of the year [7, 8]. As a consequence, the moisture stress between rainfall events (dry spells) is responsible for most crop yield reductions and sometimes even for total crop failures [9]. These events have challenged agricultural activities, especially rain-fed agriculture in Ethiopia [10]. In addition to the abovementioned factors, due to high population growth in the highland areas of Ethiopia, more and more marginal areas are being utilized, challenging agricultural productivity. Accordingly, one of the major challenges in the country is how to promote food production to meet the over increasing demand of the growing human population [9, 10].

Small-holder farmers need to be involved and consulted if any new technology or practice aimed at improving agricultural productivity is to succeed [11]. Institutions must respond to the needs of farmers ensuring reliable, efficient, and equitable access to water. This will require changes in attitude of farmers toward adoption of the technologies. Moreover, well targeted investments in infrastructure modernization, institutional restructuring, and upgrading of the technical capacities of farmers and water managers are necessary [12–15].

Technology adoption observed on store water behind dams, tanks, and ponds when water is abundant and where it can be used for times of shortage [12]. Water storage spurs economic growth and helps alleviate poverty by making water available when and where it is needed [16, 17]. There is a growing interest in low-cost agricultural water management technologies in the arid and semiarid areas of developing countries [18, 19]. One of the promising technologies to combat the problem of food insecurity in arid and semiarid lands is the use of rainwater harvesting systems [18, 20, 21]. Focusing on low-cost rain water harvesting technologies to tackle the pressure of fresh water scarcity of rain-fed agriculture is often regarded as one of the possible responses [22–24]. Making in-depth review on the related literature, it was shown that there is a dearth of information on the perception of local farmers towards water harvesting technologies. Hence, this study aimed at assessing the perception and determinants of farmers towards water harvesting technology in Raya-Alamata district of northern Ethiopia.

## 2. Materials and Methods

### 2.1. Study Area

**Figure 1:**
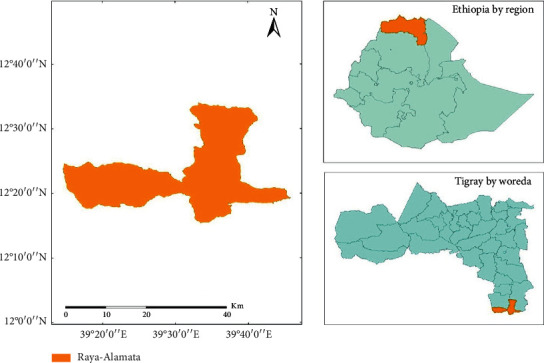
Description of the study area.

The study was carried out in a tropical district in northern Ethiopia (see Figure 1). The altitude of the area is about 1562 m above sea level, and the mean annual precipitation is about 790 mm. Rainfall is high and long from mid June to the end of August. The high potential evaporation affects crop production in the study area. Water in the study area is obtained through the means of direct rainfall, rain fall harvesting, river water, and ground water. With the recent global warming, concerns over water scarcity have increased in the study area.

Source: Mekelle University GIS Lab, 2019.

### 2.2. Sampling Methods, Data Collection, and Data Analysis

The study was conducted purposely in four kebeles (the lowest administrative units in Ethiopia) of Raya-Alamata. The four kebeles (Lmat, Selenwuha, Hulgzelemelem, and Harle) were selected purposively as they are among the kebeles with a large number of farmers using rain water harvesting technologies for crop production. In the selected kebeles, small-holder farmers (adopters and nonadopters of water harvesting technology) were deliberately considered as study samples. Sample size was proportionally determined from the total households of the four kebeles. Based on Yamane's (1967) sample size determination formula cited in [25], 270 sample respondents were considered using a proportionate stratified sampling technique. Respondents were withdrawn systematically from the list provided from each subdistrict local government office; and its proportion is shown in Table 1.

Focus group discussion, interview with district and subdistrict administrators, agriculture experts, technical crop supervisors, elders, key informants, and local administrators were used to supplement the primary data that were collected through a questionnaire. Furthermore, secondary data were gathered from manuals, policy statements, and proclamations.

Descriptive statistics and simple narration were employed to analyze the data. Descriptive statistics such as mean, percentage, and standard deviations were used to clearly stipulate the perception or attitude of local community towards rain water harvesting technology. For triangulation purpose, data collected through interview and focus group discussion were analyzed qualitatively through narration.

### 2.3. Theoretical Model and Empirical Specification

In this paper, regardless of the intensity and quantity of technologies being used, a farmer was taken as an adopter if he or she adopts water harvesting technology. The dependent variable, technology adoption, has a binary nature taking the value of 1 for adopters and 0 for nonadopters. In this regard, an econometric model employed while examining probability of farm households' rain water harvesting technology adoption decision was the probit model. Often, the probit model is imperative when an individual is to choose one from two alternative choices, in this case, either to adopt or not to adopt rain water harvesting technology. Hence, an individual *i* makes a decision to adopt rain water harvesting technology if the utility associated with that adoption choice (*V*_1*i*_) is higher than the utility associated with the decision not to adopt (*V*_0*i*_). Hence, in this model, there is a latent or unobservable variable that takes all the values in (−*∞*, +*∞*). According to [26], these two different alternatives and respective utilities can be quantified as *Y*_*i*_^*∗*^= *V*_1*i*_ − *V*_0*i*_, and the econometric specification of the model is given in its latent as

(1) $$\begin{array}{c} \left\{Y_{i}=\left\{\begin{array}{c} 1,\text{ if }y_{i}^{*}\geq 0,\\ 0,\text{ if }y_{i}^{*}<0,\; \end{array}\right.\right. \end{array}$$

where *Y*_*i*_ takes the value of one (1) for adopters and zero (0) for nonadopters.

(2) $$\begin{array}{c} Y*i=X^{\prime }\;\beta i+ui, \end{array}$$

where *u|x* is a normally distributed error term. From this unobserved or latent model specification, therefore, the utility function depends on household specific attributes *X* and a disturbance term (*u*) having a zero mean:

(3) $$\begin{array}{c} U_{i1}\left(X\right)=\beta 1Xi+u_{i0}\text{ for adopters.} \end{array}$$

As utility is random, the *i*^th^ household will adopt if and only if *U*_*i*1_ *>* *U*_*i*0_.

Thus, for the household *i*, the probability of adoption is given by

(4) $$\begin{array}{c} P\left(1\right)=P\left(U_{i1}>U_{i0}\right),\\ P\left(1\right)=P\left(\beta 1X1+\;u_{i1}>\beta 0Xi+\;u_{i0}\right),\\ P\left(1\right)=P\left(u_{i0}-u_{i1}<\beta 1\;Xi-\beta 0\;Xi\right),\\ P\left(1\right)=P\left(ui<\;\beta Xi\right),\\ P\left(Y_{i}=1\right)=\varphi \left(\frac{-X^{\prime }i\beta }{\sigma }\right), \end{array}$$

where *P*(1) is the probability of adopting rain water harvesting technology. *φ* is the cumulative distribution function of the standard normal distribution. *β* is the parameters that are estimated by maximum likelihood.

*x*′ is a vector of exogenous variables that explains adoption of rain water harvesting technology (e.g., age of the household head, sex of the household head, education, and access to credit). Therefore, on the basis of the dependent variables indicated, rain water harvesting technology, the probit model was applied for the binary dependent variable as follows:

(5) $$\begin{array}{c} \text{RWHTA}{=}_{\delta 0}+{\;}_{\delta 1}\text{AGE}{+}_{\delta 2}\text{SEX}{+}_{\delta 3}\text{EDUCA}{+}_{\delta 4}\text{HHSIZE}{+}_{\delta 5}\text{FARMS}{+}_{\delta 6}\text{LOCA}{+}_{\delta 7}\text{OFF}-\text{INCOME}{+}_{\delta 8}\text{DISFTC}{+}_{\delta 9}\text{DFARM}+\varepsilon_{i}, \end{array}$$

where RWHTA is a dependent variable indicating for probability of rain water harvesting technology adoption. Given the abovementioned dependent variable (rain water harvesting technology), to estimate the magnitude of parameters or variables basically to put clearly the percentage probability of adoption, marginal effect of variables was calculated (see Table 2 for marginal effect results). Marginal effect of a variable is the effect of unit change of that variable on the probability of *P*(*Y*=1*|X*=*x*), given that all other variables are constant. The marginal effect is expressed as

(6) Yi=1/Xi∂X i=Yi/Xi∂X i=Xi′βφβ.

The probit model specified above estimates the probability of households' adoption of rain water harvesting technology. The explanatory variables, unit of measurement, character, and expected signs are described in Table 3.

## 3. Result and Discussion

### 3.1. Perception of a Farmer toward Rain Water Harvesting Technology

Understanding the perception of the community is pertinent basically for making strategies and development endeavors sustainable and to communicate with the entire community to ensure a sense of belongingness. Primarily, the study observed that it was mostly men who participate and are active in the rain water harvesting system as compared to the women in the four kebeles. Probing further, through the responses received from the interviewees when asked why more men are engaged in this technological activity than men, they mentioned the fact that men are mostly responsible in farming as compared to women. One female participant in Harle responded saying “Though I am a user of water harvesting, it is mostly my elder son who engages in the group activities in addition to other farm activities together with my hired laborers.”

According to the view of focus group discussants, water harvesting and making it community based is useful as source drinking for their animals, for boosting rainfall, as a means to protect land degradation and maintaining soil fertility and to grow timber and fire wood. Moreover, this perception was further strengthened by their view that benefit from rain water harvesting do ranges from satisfying basic domestic needs to the extent of providing authority or power and, thereby, creating a sense of belongingness. On the other hand, some focus group discussants and interviewees have claimed that these advantages and sense of belongingness were not in place due to tenure insecurity. Hence, such advantages would become real after the devolution of any control system from the state, making the technology community based and, thereby, community owned.

While measuring the view of sample respondents towards rain water harvesting technology and the importance of participation, around (74%) of them have perceived and rated it as “Very Well.” Farmers have their own rain water harvesting management norms that can be considered as local institutions. As have already been purported by Oremo et al. [27], institutions are a set of complex norms regulating the action of persons in the process of social interaction, representing local systems of authority derived from sociocultural and historical processes of a given society.

On the basis of the key informants' interview carried out with local leaders in Hulgzelemlem, rules, regulations, and sanctions of local institution do totally forbid using water for farm purpose without request. They are allowed only for drinking by their livestock during dry seasons. This clearly shows that the community is highly aware about the significance of avoiding extravagant practices. The finding is in line with the study in [28] which highlighted the importance of collective action of local institutions in managing common resources such as water.

Table 2 further indicates that, around (82%) of the respondents have agreed that local institutions are enhancing factors of participation in managing water resources. This might be due to the fact that local communities do respect rules and regulations of the indigenous institution. The rules are formulated by themselves based on their own living conditions and contexts without external intervention. Practicing leadership in local institutions and managing local resources are clear manifestations of decentralization, paving the way for fair distribution of water and conflict resolution. Furthermore, about 25% of the respondents were found to be participants in decision making, who guided the other members to participate in the activities (pond construction and filling the ponds during the rainy season). Those who participate in management protect the water using fencing, roof, and through funding/financing the salaries of guards. On the other hand, focus group discussants and interviewees indicated that shortage of fence and roof construction materials and insufficient rain fall pose frequent challenges.

### 3.2. Determinants of Farmers' Decision Adopting Rain Water Harvesting Technology

The statistical results of this study are presented Table 4. The significant variables are discussed below.

The econometric result was consistent with the prior expectation that sex would influence farmers' adoption of the technology. This variable was statistically significant at 10% indicating that males have 40% higher likelihood of having a better level of adoption than their counterparts. Females' share of adoption of the technology was very low partly due to their productive and reproductive roles. Information from some women respondents during focus group discussion and interview revealed that multiple burdens such as childcare, cooking, and travel to long-distance markets present major challenges of technology adoption.

The econometric result for education indicated that the researcher's expectation of the influence of education on RWHT is positive, consistent, and significant as well. In this study, as the educational level increases by one, probability of adoption gets higher by 4%. Obviously, education is an input in awareness creation about the new technologies.

According to the information from the survey, more than 85% of the small-holder farmers have their own land. This variable was statistically significant at 10%, showing that a one unit increase in farm size raises the likelihood adoption by 94%. Because the larger the farm, the more likely a farmer becomes to construct a rain water harvesting pond on his/her plot of land. On the contrary, small plots of land make constructing a pond more difficult. This is supported by data in the study showing that, irrespective of their level of awareness, as farm size decreases by one unit, the level of adoption decreases by 6%.

It is also not surprising to find that households that get more benefit from off-farm income are more likely to adopt the water harvesting technology. Households in a community become more justified to construct a pond when they have better off-farm income. Higher economic return from off-farm income encourages them to buy motor pumps, fence, and roof covers. The other important constraint identified in the study area is lack of materials and equipments in the utilization of stored water. The stored water in the ponds needs to be lifted to be used. Most farmers use buckets to lift water. An interview with Raya-Alamata agriculture and rural development office experts indicated that, for some of the early adopters, water lifting buckets were supplied at lower prices. However, as stored water decreases, lifting becomes more difficult (especially for handicap and female farmers). This calls for alternative means such as using motor pumps which most farmers do not afford.

Distance to farmers' training center has a negative effect on the likelihood of a household adoption of RWHT. There was a significant difference between the average adopters' and nonadopters' home distances from FTC. As the distance of the home from the FTC is increased by a kilometer, the probability of the farmer's adoption to RWHT decreased by 25%. This might be due to time delay and information asymmetry for residents farthest from the FTC.

The variable distance to the farm was also found to be significant in determining the level of adoption. On average, a household near to the farm has 12% more likelihood of becoming a high-level adopter compared to the farmers whose farms were far from home.

## 4. Conclusions and Recommendations

The study revealed that majority of the respondents have perceived that rainwater harvesting technology is pertinent in improving farm income, regenerating forest, and encouraging the right to use water resources. Furthermore, the farmers use water resources, especially during the dry season, for their animals with permission from local leaders. The results revealed that rainwater harvesting technology is seen by the community members to be a good initiative in improving agricultural practices in periods of water scarcity. Some of the perceptions of the community against the technologies were labour intensity, lack of technical know-how and need for extensive training, and information asymmetry.

The probit regression models revealed that determinants of farmers' adoption of rainwater harvesting technology were significantly and positively affected by education, farm size, and off-farm income. The effect of the distance to the farmland and farmers training center (FTC) was also significant but negative. Farmers' adoption towards rainwater harvesting technology was also significantly determined by sex, with men farmers having higher level of adoption compared to their counterparts. Productive and reproductive roles constrain women household farmers from using the technologies.

To raise the level of perception, we recommend the dissemination of rain water harvesting technology-related information through formal and informal education. Alternative sources of water such as ground water and river water need also be promoted. To protect the water resources from damage, a strong and sustainable water management policy should be enforced. More importantly, female-headed households need to be encouraged to be community leaders to foster the adoption of rain water harvesting technology.

## Figures and Tables

**Table 1:** The targeted kebeles, their respective household numbers, and the sample size to be taken from each kebele (sampling frame) using a proportionate stratified sampling technique.

Name of selected kebeles	Total household heads	Number of respondents by sex		Total sample size of each kebele	Type of sampling (probability)
		Male	Female		
1. Lmat	1697	33	32	65	Stratified and systematic
2. Selenwuha	2474	50	45	95	Stratified and systematic
3. Hulgzelemlem	1756	34	34	68	Stratified and systematic
4. Harle	915	20	22	42	Stratified and systematic
Total	6842	137	133	270	

Source: own computation, 2019.

**Table 2:** Subdistrict households' perception towards water harvesting technology and their level of participation.

Variables	Labels	Frequency	Percentage
Perception on RWHT and importance of participation	Very well	199	73.7
	Moderate	51	18.9
	Low	20	7.4
	Total	270	100

Role of local institutions in RWHT	Being initiator	222	82.3
	Being inhibitor	32	11.8
	No effect	16	5.9
	Total	270	100

Participation approaches in RWHT	Decision making	68	25.2
	Activities	118	43.7
	Management	84	31.1
	Total	270	100

Source: author's survey result (2019).

**Table 3:** The explanatory variables, unit of measurement, character, and expected signs.

Variable type	Character	Expected direction with the level of adoption
1. Household head age (AGE), years	Continuous	+
2. Sex of the house hold heads	Dummy (0, female and male, 1)	+
3. Level of education (EDUCYRS), years	Continuous	+
4. Size of the household (HHSIZE)	Continuous	+
5. Farm size	Continuous	+
6. Location/topography	Dummy (0, steep slope and 1, plain)	−
7. Off-farm income	Continuous	+
8. Distance FTC	Continuous	−
9. Distance of the farm land	Continuous	+

Source: author's survey (2019).

**Table 4:** Econometric modeling.

Probit regression		Std. err	*z*	*P* > |*z*|	Number of obs = 270	
Log likelihood = 147.8284					LR chi2 (9) = 78.27	
					Prob > chi2 = 0.0000	
					Pseudo *R*2 = 0.2093	
rwhta	Coef.				(95% conf. Interval)	
Sex	0.4038505	0.1916587	2.11	0.035^*∗*^	0282063	0.77949476
Age	0.0061611	0.0059873	1.03	0.303	−0.0055739	0.017896
Edu	0.040628	0.0232639	1.75	0.081^*∗*^	−0.0049685	0.0862245
Fams	0.0311723	0.0421629	0.74	0.460	−0.0514655	0.1138101
Fars	0.9416275	0.5332914	1.77	0.077^*∗*^	−0.1036045	1.986859
Location	0.0298328	0.2145869	0.14	0.889	−0.3907499	0.4504155
Offin	0.0009064	0.0002596	3.49	0.000^*∗∗∗*^	−0.0014152	−0.0003976
Dftc	−0.2583337	0.0550596	−4.69	0.000^*∗∗∗*^	−0.3662485	−0.1504189
Dfarm	−0.1211989	0.0639297	−1.90	0.058^*∗*^	−0.2464989	0.0041011
Cons	0.3471292	0.3965447	0.88	0.381	−0.4300842	1.124343

Source: Stata 12 output and authors' survey (2019), ^*∗*^, ^*∗∗*^, and ^*∗∗∗*^: significant at 10, 5, and 1%, respectively. SE = standard error; MREF = marginal effect.

## Data Availability

The original data generated during the study and used in this paper are available with the corresponding author and can be provided on reasonable request.

## Supplementary Materials

Supplementary Materials The Supplementary Materials contain the questionnaire which is prepared by a researcher in Mekelle University for a partial small-scale grant. The aim of this questionnaire is to collect data about “How Small-Scale Farmers Understand Rain Water Harvesting Technology? Evidence from Northern Ethiopia.”
