# Supplementary material for: How Small-Scale Farmers Understand Rain Water Harvesting Technology? Evidence from Northern Ethiopia
Source: ScientificWorldJournal. 2021 Jan 28;2021:8617098. doi: 10.1155/2021/8617098 (PMC7861931; doi:10.1155/2021/8617098)
Supplement: Supplementary Materials — The Supplementary Materials contain the questionnaire which is prepared by a researcher in Mekelle University for a partial small-scale grant. The aim of this questionnaire is to collect data about “How Small-Scale Farmers Understand Rain Water Harvesting Technology? Evidence from Northern Ethiopia.” [file 8617098.f1.docx]

Mekelle University

College of Dry land Agriculture and Natural resources

Department of Rural Development and Agricultural Extension

Household Survey Questionnaire to be Responded by Farm Households

Introduction:

This questionnaire is prepared by a researcher in Mekelle University for partial small scale grant. The aim of this questionnaire is to collect data about “HOW SMALL-SCALE FARMERS UNDERSTAND RAIN WATER HARVESTING TECHNOLOGY, EVIDENCE FROM NORTHERN ETHIOPIA?”. The information you provide is believed to have a great value for the success of this research. I confirm you that all data will be used for academic purpose and will be analyzed anonymously and you are not exposed to any harm because of the information you give. I highly appreciate in advance to your kind cooperation in providing the necessary information.

Thank you!

General instruction:

1. Please choose appropriately represents your response from the multiple choices

2. To the open-ended questions, please write your response on the space provided.

Part one personal and Physical factor

1. Kebelle (location)-------- 2. Age ------ 3. Gender -------4. Level of Education….....

5. Family size---------

6. Distance home from market in hours -----------

7. Distance home from the farmland in hours------------ 7.1 farm size__________

Part two Determinants of Farmers Decision Participation in Rain Water Harvesting Technology

8. What do you think currently about Rain Water Harvesting Technology in your locality?

1) it is common

2) it is rare

3) There is no Rain Water Harvesting Technology at all

9. What is the major water source for you, your farm and your animals?

1) river water 2) ground water 3) rain water

4) Other (Specify)…………………………………

10. If your answer for Q. No 8 is “it is common”, are adopter or non adopter rain water harvesting technology? If you are adopter why? ---------------------------------------------------------------------------------------------------------------------------------------------------------------------------------------------------------

11. Do you have any knowledge about the Rain Water Harvesting Technology?

1) Yes 2) No

12. If yes what are these?-----------------------------------------------------------------------------------------------------------------------------------------------------------------------------------------------------------

13. How do you know?

1) Formal learning in school 2) Information from mass media

3) Informal learning through training 4) others, (Specify)…………….

14. If your answer for Q. 11 is yes, what is its impact from your experience?

1) It has strong positive impact on adoption.

2) It has less positive impact on adoption

3) no impact at all

15. What are your main energy source for lifting water sources from the damp?

1) manually human power 2) wind energy 3) generator 4) Electricity 5) Other (Specify)…………………………………

16. Who help you to construct pond for water harvesting ? (Arrange them in order)

1) Government 2) Individuals 3) NGOs

4) Other stakeholders 5) Others (specify) ------

17. Do you agree Rain Water Harvesting Technology increasing crop production?

1) Strongly agree 2) agree 3) disagree 4) strongly disagree

28. If your answer for Q.17 is options 1 and 2, what could be the reasons?

1) It gives Authority (power) to the community

2) It create feeling a sense of belongingness

3) There is increment of forest regeneration

4) Encourage the right to use water

5) Others (Specify)……………….

19. Who should manage ponds? (Please rank them in order of importance)

1) All stakeholders

2) Government and all users

3) Immediate users at local level

4) Community and NGO‟s partnership

5) Indigenous institutions

6) Other (Specify) ……………………….

20. Do you believe that the current land tenure in Ethiopia has a link (any relation) with constructing water pond?

1) Yes 2) No

21. If your answer for Q. NO 20 is yes, how do you rate the degree of its effect on your

adoption?

1) Strongly enhance 2) Slightly enhance

3) Strongly hinder 4) slightly hinder

22. Do you think that the adoption of Rain Water Harvesting Technology has short coming ?

1) Yes 2) No

23. If yes to Q. No 22, what are the major shortcomings? --------------------------------------------------------------------------------------------------------------------------------------------------------------------

24. If No for Q. No 22, what is your reason as adoption of Rain Water Harvesting Technology has short has no shortcomings? ---------------------------------------------------------------------------------------------------------------------------------------------

25. What is the status of crop production around your living area after the introduction of Rain Water Harvesting Technology?

1) Decreasing 2) Increasing 3) no change

26. If your answer for Q. No 25 is 1 or 2, what are the major reasons? --------------------------------------------------------------------------------------------------------------------------------------------

27. Have you ever been aware about Rain Water Harvesting Technology? 1) Yes 2) No

28. If your answer for Q.27 is yes does this initiate you to participate actively?

1) Strongly 2) slightly 3) no effect

29. Do get any benefits from Rain Water Harvesting Technology?

1) Yes 2) No

30. If your answer for Q.29 is yes do these initiate you in adopting Rain Water Harvesting Technology?

1) Strongly 2) Slightly 3) Nothing

31. What is the interest of the community in your village looks like on the issue of water resource management?--------------------------------------------------------------------------------------------------------------------------------------------------------------------------------------------------------------

3.2. Institutional factors
32. Are there local institutions in your locality to manage water resources ?

1) Yes 2) No

33. If Yes for Q. No 32, what is their effect in participation of the people?

1) They are initiators 2) They are inhibitors 3) Nothing

34. What is the cultural value or belief of the community towards Rain Water Harvesting Technology?

1) reduce water from misuse

2) Sustainable use of water resource

3) Using as much as needed for personal consumption

4) There is no relation with water

35 .Does cultural value has any impact on your participation?

1) Yes, it encourages 2) Yes; it discourages 3) No impact

36. How is the degree of enforcement of rules and regulations of an association?

1) Very high 2) high 3) low

37. How do you see local administration in association rules and regulations enforcement?

1) Very high 2) high 3) low

38. If your answer for Q. No 37 is yes, it encourages state the relationship between the value and

participation-------------------------------------------------------------------------------------- ---------------

3.3. Economic factors

39. What is your main source of income and household activities?

No Activities Tick

1 Crop production

2 Livestock production, 39. 2.1 How much domostic do you have list them__________

3 Fuel wood selling

4 other off farm activities

40. How much do you earn annually__________

40.1 how much do you earn from nonfarm activity___________

41. What is the income distribution within the group you belong to looks like?

1) Significant differences 2) Slight differences 3) Fair distribution

42.Do you believe that the income difference causes participation differences in water harvesting technology?

1) Yes 2) No

43. If your answer for Q.42 is yes, answer the following

1) That higher income group participates actively than that lower income group

2) Those lower income group participate actively than those higher income groups

3) Those medium income group participate actively than those lower and higher incomes

44. What are the major factors that enhance yours participation in rain water harvesting technology?

Please list down by priorities ------------------------------------------------------------------------------------------------------------------------------------------------------------------------------------------------------56. What are the major factors that hinder you rain water harvesting technology? Please list

down by priorities? ------------------------------------------------------------------------------------------------------------------------------------------------------------------------------------------------------ -----------
